# Supplementary material for: Asynchronous magnetic resonance elastography: Shear wave speed reconstruction using noise correlation of incoherent waves
Source: Magn Reson Med. 2022 Oct 27;89(3):990–1001. doi: 10.1002/mrm.29502 (PMC9792433; doi:10.1002/mrm.29502)
Supplement: Supplementary file 1 — DATA S1 MATLAB code used to generate the numerical simulations of Figure 1 [file MRM-89-990-s002.zip › k-Wave/helpfiles/k-wave_time_varying_source_problems.html]

Time Varying Source Problems (k-Wave)


# Time Varying Source Problems

- Example: Monopole Point Source In A Homogeneous Propagation Medium
- Example: Dipole Point Source In A Homogeneous Propagation Medium
- Example: Simulating Transducer Field Patterns
- Example: Steering A Linear Array
- Example: Snell's Law And Critical Angle Reflection
- Example: The Doppler Effect
- Example: Diffraction Through A Slit
- Example: Simulations In Three Dimensions
- Example: Simulating CW Fields Using The Acoustic Field Propagator
- Example: Holographic Projection Using The Angular Spectrum Method
- Example: Equivalent Source Holography
